# Food Improvement Goals in Schools (FIGS I): a qualitative evaluation of a whole-school approach to healthy food provision in primary schools

**DOI:** 10.1186/s12889-026-27503-0

**Published:** 2026-04-22

**Authors:** Natalia Concha, Maria Bryant, Carol Dezateux, Katy Scammell, Rab Brownell, Mathilda O’Donoghue, Pratima Singh, Meredith K.D. Hawking

**Affiliations:** 1 Centre for Primary Care, Wolfson Institute of Population Health, Queen Mary University of London, 58 Turner Street, London, E1 2AB UK; 2 The Hull York Medical School and Department of Health Sciences, University of York, York, YO10 5DD UK; 3 Public Health Division, London Borough of Tower Hamlets, 160 Whitechapel Road, London, E1 1BJ UK; 4 Healthy Lives, London Borough of Tower Hamlets, 160 Whitechapel Road, London, E1 1BJ UK; 5 Centre for Public Health and Policy, Wolfson Institute of Population Health, Queen Mary University of London, 58 Turner Street, London, E1 2AB UK

**Keywords:** Fee school meals, Food insecurity, Universal free school meals, Whole-school approaches to food, Children, Health inequalities, Qualitative evaluation, Focussed ethnography, School food improvement, Elementary schools

## Abstract

**Background:**

Free school meals offer a crucial safety net for children facing poverty and food insecurity. In the London Borough of Tower Hamlets in England, where over half of local children experience poverty, child-centred policies like universal free school meals have been implemented to support all children, regardless of family income. Despite World Health Organization recommendations for whole-school approaches to food, many schools have yet to implement them. The Food Improvement Goals in Schools I study, funded by ActEarly, evaluated the “Fantastic Food in Schools” programme, aiming to improve whole-school approaches to healthy food, including universal free school meals, in Tower Hamlets’ primary schools.

**Methods:**

Informed by material, relational and symbolic factors, we explored how food provision and interventions were perceived and practiced in six primary schools. A focused ethnography was conducted over 210 h across seven months in 2023, involving observations of interventions, sharing lunches with children and over 100 informal conversations. We carried out semi-structured interviews with 16 staff, 20 parents and 12 mini-groups with 43 children, using creative methods. Data were analysed thematically and interpreted through panels with community researchers.

**Results:**

Universal free school meals were perceived to promote equity and health, particularly given the UK cost-of-living crisis. Child-centred interventions engaged children and families, encouraging healthy eating at school and at home. Schools served as spaces to explore food’s cultural significance, helping navigate the complexities of children’s preferences, familiarity, identities, healthier eating and needs in a diverse community. School menus improved, given collaborative efforts from catering services and kitchen staff. Yet, resource challenges and growing parental expectations to address systemic issues like food insecurity highlight the need for greater funding and policy-based support.

**Conclusions:**

Universal free school meals and whole-school approaches to food can enable equitable cultural inclusion in diverse, urban areas like Tower Hamlets, supporting *Sustainable Development Goal 2: Zero Hunger*. However, these initiatives require increased funding and targeted financial incentives for schools to implement them effectively. Local authorities need the backing of educational policy to prioritise and secure the necessary resources to promote healthy eating and ensure equitable access to nutritious food for all children.

**Supplementary Information:**

The online version contains supplementary material available at 10.1186/s12889-026-27503-0.

## Background

Food insecurity, defined by the United Nations as a lack of access to sufficient, safe and nutritious food for a healthy life, is a multifaceted and growing issue in high income countries. In the United Kingdom (UK), over four million children live in households experiencing food insecurity [1–3]. For many families, food insecurity equates with a ‘hand to mouth’ existence [4]. The recent UK cost-of-living crisis has further exacerbated this situation, with an estimated one million children living in destitution [5]. Children living in these conditions often lack daily essentials and face hunger, all of which can detrimentally affect their mental and physical health [6–9]. Socioeconomic inequalities also increase the risk of diet-related conditions, including childhood obesity, dental caries and malnutrition. Children living with overweight and obesity face a higher risk of carrying excess weight into adulthood, increasing risks of type 2 diabetes, cardiovascular disease, cancers and long-term health conditions [10–12], as well as a range of adverse outcomes with childhood onset [13–15]. Given the vital role food insecurity plays in impacting children’s health and development [6, 7, 16–19], this crisis reinforces the need to support children’s right to healthy and nutritious food, demanding strategies that align with the United Nations Convention on the Rights of the Child [20].

### The policy response: universal free school meals (UFSMs)

School feeding programmes now provide the largest social safety net globally, feeding 418 million children during every school day in 2022 [21]. They are found to reduce food insecurity and enhance children’s education and health, as part of global efforts to achieve *Sustainable Development Goal 2: Zero Hunger.* By offering free school meals (FSMs), these programmes provide a critical support system for children facing poverty and inequalities [21, 22]. In addition to mitigating food insecurity, a systematic review of 47 studies evaluating universal free school meals (UFSMs) emphasised subsequent improvements in student participation, nutrition and academic performance [16], and found they are a cost-effective investment for children and young people worldwide [16, 17]. Given that children typically consume about a third of their dietary intake at school, UFSM programmes have the potential to improve child outcomes.

In England, UFSMs are offered to all children in their first three years of primary (elementary) school (aged 4–7 years old) in state-funded schools. Beyond this, almost 20% of school-aged children qualify for means-tested FSMs based on their household income and receipt of parent/carer welfare support, which results in indirect financial benefits to the school in the form of a pupil premium. However, 800,000 children living in poverty were excluded from FSM entitlement under the means-tested system in place during the study’s period (2023–2024). The FSM strict eligibility criteria [1] had been restricted to children in households receiving means-tested welfare benefits known as Universal Credit, with a household net income below £7,400 a year, equivalent to $9,890 USD (September 2024 conversion rates) [23]. Many families experiencing food insecurity and poverty have been ineligible for FSM under this earnings cap [24]. These narrow eligibility cut-offs have been widely criticised for failing to address the social gradient of disadvantage, where families just about the threshold may be experiencing poverty [25]. Within this policy context, reliance on the reduced means-tested offer has entailed multiple barriers, including information and practical barriers centred on access [26, 27], as well as concerns over food quality and choice [28] but others also reveal the importance of stigma and peer dynamics [29–31]. Since this study was conducted, the UK Government has announced an expansion of FSM eligibility to all children in households receiving Universal Credit (removing the £7,400 earnings cap) from the 2026/27 school year (September 2026 onwards) as part of its Tackling Child Poverty Strategy [32]. This has been a widely welcomed policy by public health and child advocacy sectors but the Universal Credit eligibility continues to reflect a threshold-based approach that does not fully address the social gradient of health disadvantage [25]. Families experiencing in-work poverty but not receiving Universal Credit may remain excluded and benefit-based policy responses are unlikely, on their own, to lift many low-income households above a minimum acceptable standard of living, particularly in the UK cost-of-living crisis [33]. UFSMs remain a broader strategy to reduce inequalities and support children’s health and wellbeing across the income gradient [24, 25, 30, 34].

In some localities, policy responses have moved beyond means-tested provision for more than a decade [35, 36]. In the London Borough of Tower Hamlets, where this study took place, the local authority extended the UFSM offer to include all primary school children in years 3–6 (ages 7–11 years) since 2014. Additionally, in 2023, the Mayor of London extended the provision of UFSMs to all children in the city’s primary schools [37]. Tower Hamlets is unique in that it is the only local authority in England to go further and extend UFSMs to secondary schools since 2023, offering UFSMs for all primary aged children and young people aged 4–16. This long-term provision, covering twelve consecutive years, provides sustained access to UFSMs throughout critical developmental periods, from childhood into adolescence.

### Factors influencing school food provision

The economic and social protection benefits of UFSM schemes for children experiencing food insecurity and poverty are clearly documented [27], but there is also growing emphasis on improving nutritional quality among nutritionists, public health, and catering organisations [38]. School food provision is complex and influenced by multiple drivers and barriers [27, 38–40], particularly in economically deprived areas. For instance, recognising the importance of children’s food preferences and needs in intercultural schools entails accommodating dietary requirements, quality and variety, incorporating faith-based, cultural [41, 42] and children’s own preferences, alongside appropriate portion sizes and maintaining food standards [28, 43]. Beyond the food itself, non-food related aspects are also key. These include material, spatial, and time constraints [44, 45], as well as the social and developmental significance of school spaces [46, 47], including peer group interactions and a sense of belonging [28, 44, 48–50]. Broader organisational challenges within schools such as staffing and funding can also be heavily impacted by macro-level factors [39].

Within this context, we recognise the active role that children play in decision-making around school food provision. Children voice their preferences for variety, quality, quantity and choice between packed lunches or school meals [28, 43, 51]. Their choices are also shaped relationally by parents, carers, family or friends, kitchen and school staff, as well as social media [52]. Together, these influential attitudes, norms and practices may shape the meals that children choose to eat at school [28, 43, 51, 53–56].

School food provisioning models are recommended to adopt whole-school approaches to food, based on the World Health Organization (WHO) framework integrating health and education within the school environment through policies, curricula, community links and to improve and sustain broader health outcomes (WHO, 1997). These approaches have been advocated for UK school food interventions seeking system transformation, by looking at culture, policies and accessibility [39, 57, 58]. This creates supportive environments where children develop the agency to make healthy choices, not just through education but also through their daily experiences and the food culture they encounter [59]. Such strategies have the potential to bring food education outside of the classroom, influencing broader eating habits and behaviours, while also moving away from an individualistic focus on nutrition, by promoting collective agency for healthy food practices across the school community [59–61]. However, faced with multiple other priorities, schools rarely implement whole-school approaches to food.

### Research setting: the Food Improvement Goals in Schools (FIGS I) study

The FIGS I study was a focused ethnography evaluating primary school-based food provision within a recent initiative: “Fantastic Food in Schools” (FFiS), a three-year local government led programme in the London Borough of Tower Hamlets. The FFiS programme was designed to respond to the area’s high levels of child poverty, food insecurity and childhood obesity [62]. FFiS adopted a whole-school approach to food, aiming to provide nutritious meals and deliver food education by inculcating a wider school food culture in primary schools.

### Aim and research questions

The evaluation aims of FIGS I were co-designed with academics (MB, CD, MH and NC) and members from Tower Hamlets Council public health team (KS, RB), focussing on key questions linked to a logic model to inform the FFiS programme.

#### Aim

To explore the current conditions (perceptions, practices and resources) of school food provision for primary school-aged children to support the qualitative evidence-base informing the Council’s recent FFiS initiative.

#### Research questions

1. What are the factors that influence the degree to which the programme has the potential to improve equitable healthy eating and the uptake of healthy whole-school approaches to food?
2. Is the programme delivered as intended? How is the programme received? What is the experience of its acceptability and feasibility?
3. What are the perceived changes (if any) of pupils, and to what extent (and how) has the programme changed healthy eating, and delivered wider social value benefits equitably?

## Methods

### The FFiS programme

The programme aimed to improve the nutritional content of food provided in schools, maximising the health benefits from funding universal free school meals, while creating an environment where children enjoy, and benefit from, eating healthily. The main elements of the programme and the education and engagement interventions are described in Table 1 below:

**Table 1:** Educational and engaging interventions delivered by the FFiS programme

Main elements of the FFiS programme
• Grant principles and enhanced school food standards: schools are expected to implement standards including a healthy dessert and water-only policy.
• Council support to schools to develop and implement a whole-school food approach to healthy eating.
• Collaboration with the Council’s central catering service (covering approximately 70% of schools in the borough) to design new menus, conduct audits and train school kitchen staff.
• The Council’s Healthy Lives team further supports schools in achieving Healthy School Awards at bronze, silver and gold levels, enhancing meal quality, nutrition and variety through collaboration with the catering service.
FFiS Programme Activities
• TastEd (Taste Education) sessions: Sensory, hands-on learning sessions aimed at broadening children’s exposure to healthier food options, particularly fruits and vegetables.
• Cook-a-thon: An interactive competition event where children engage in cooking and tasting healthy foods, designed to encourage learning about nutrition in a fun, playful way.
• Parent tasters: Parents were invited to taste school meals and engage in discussions around menu options.
• Family-style dining: A shared dining experience where children serve themselves and eat together with teachers or wider school peers.

### A focused ethnography with a community health psychology lens

The study draws on a community health psychology framework [63, 64] unpacking the material, relational and symbolic factors shaping school food provision, applying a focused ethnography to capture the lived experiences of children, parents and staff. This method is well-suited for time-limited, data-intensive applied research projects with short-term site visits [65]. It enabled a closer encounter with participants, guided by participatory principles [66] and helped to address issues of trust with excluded communities given the historic asymmetrical power hierarchies [67]. The focused ethnography also facilitated multiple data elicitation methods, which are valuable in intervention research with children [68].

### Sampling / recruitment

We worked with Tower Hamlets Council partners (KS and RB), headteachers and staff to navigate access to participating schools, using various school-supported routes. We applied a purposive sample to encompass ethnic diversity, faith-based and school sizes, recruiting six primary schools in Tower Hamlets over a seven-month period between May and December 2023. Parents/carers and their children were recruited through school communication channels (e.g. newsletters, emails, and personal communication) and those interested were invited to contact the research team or school staff. We had telephone conversations, texts and email exchanges to discuss the study with potential parent/carer participants. School staff recruitment was facilitated by senior staff at schools through internal channels. Adult participants were offered a choice of interview location according to preference, including at schools, at the university, remotely or in participants’ homes. The final sample size was guided by Malterud, et al.’s (2016) concept of information power, which emphasises that a study’s capacity to address its research questions relies on adapting its components (aims, guiding theory, sample specificity, quality of data generated and analysis) to the study’s purpose, focusing on the co-generated ‘quality of dialogue’ rather than reaching saturation [69]. Our participatory and flexible approach guided by information power meant that, given the depth of engagement and data quality we had gathered, we agreed to close fieldwork after collecting data in six schools.

### Fieldwork

Data collection involved over 210 h of fieldwork, including participant observations, semi-structured interviews with 16 school staff, 20 parents (19 mothers and one father), and 12 creative mini-groups with 43 children.

Our team comprises two community researchers (PS, MOD), who were formally trained prior to recruitment in research and analysis by qualitative researchers (NC, MH). The fieldwork team (NC, MH, PS, MOD) worked closely together during the focused ethnography, enabling applied and ongoing research capacity building for community researchers in the field. In turn, community researchers enriched the project with their local knowledge, background and experience in participatory research and community engagement.

#### Semi-structured (face-to-face) interviews

Our interview approach was designed to foster an environment where participants could talk openly about what matters to them [70]. Our flexible topic guides covered the school food offer; children/families’ eating preferences and practices, and programme outcomes (see Additional file 1). Most were conducted at schools (with the exception of one parent in a household and one parent in our university office, as per their preference). Interviews lasted between 27 and 65 min and were audio-recorded.

#### Observations 

Observations of school mealtimes (lunch time, breakfast/afterschool club snacks), included sharing lunches with children. Fieldwork researchers (NC, MOD, PS and MH) sat and ate school meals with children on 26 occasions. This enabled us to experience the food and mealtime setting first-hand, by directly sharing with children not just the food but the everyday rhythms and interactions of school mealtimes. We participated in more than 100 informal conversations with staff, children and parents during school visits. We observed 27 FFiS activities: TastEd, Cook-a-thon, parent taster sessions and family-style dining. These were documented in reflexive fieldnotes guided by a template (see Additional File 1), which captured the everyday practices and dynamics of the school food environment and our interactions within it.

#### Mini-groups with children

Twelve creative mini-groups were conducted with a total of 43 children on school premises during term-time. We used visual methods applied to food [71], enriching data elicitation beyond verbal reporting. Creative tools, including visual narratives with drawings, enabled us to elicit children’s preferences, experiences and symbolic associations on school food, eating practices at home, as well as their views on the programme interventions and menu offer (see Additional File 1). While the specific activities used in our mini-groups were not formally validated, we tested and refined them through public involvement and engagement sessions with local primary-aged children prior to fieldwork. Details on this piloting process are described below.

### Public involvement and engagement

Four community engagement events were led by community researchers (PS, MOD) in collaboration with a local third sector organisation. These shaped our research priorities, building capacity, enabling ongoing research validation with schools and families, and, ultimately, capturing the lived realities and perspectives of many local families. For instance, based on children’s interactions and feedback, we adapted our data collection methods to integrate two activities into one: incorporating a short vignette [72] with a drawing exercise (a visual storytelling) to enrich participation [73]. Based on this testing of children’s exercises and on further discussions with schools and council partners, our creative activities were found to be appropriate for their ages. As a communicative validation exercise [74], we shared preliminary findings in a well-attended public panel event alongside parent volunteers and council partners, where parents particularly highlighted how our findings largely resonated with their lived experiences.

### Ethics

This study was approved by Queen Mary University of London’s Joint Research Management Office (JRMO) Ethics Panel (reference QMERC22.349). Participants were briefed and given information sheets detailing the study, voluntary participation, withdrawal rights, confidentiality, data management and permission for audio recording and dissemination. Interested participants signed consent forms (adults), parents signed consent forms on behalf of their children, and the children also gave signed assent. Research on children’s assent varies by experience and communication [75] but providing children with their own form recognises their right to choose to take part independently [20, 76]. We adhered to the university institutional policy following a detailed data management plan for quality assurance, organisation, data sharing, security and storage.

### Children’s safeguarding

Following the UN’s Convention on the Rights of the Child [20], we adopted a child-centred approach to safeguarding [77]. The fieldwork team conducted UK mandatory Enhanced Disclosure and Barring Service (DBS) checks, required for working with children. We complied with participating schools safeguarding requirements and worked in pairs during field visits.

### Data analysis

Data from the interviews and groups were transcribed verbatim, and analysis was undertaken by our research team (NC, MH, MOD, PS), complemented by reflexive fieldnotes [78], and coded using NVivo14 software. Thematic analysis was based on identifying common and divergent patterns, while maintaining their relevance to the research questions [79]. Coding developed organically, following an iterative or abductive approach to thematic analysis, combining inductive and deductive reasoning [80, 81], based on Braun and Clarke’s (2006) classic guidance. Two orders of analysis were applied: a first order mapped out salient and distinctive codes through a data reduction topical exercise (i.e., perceptions, preferences and practices around UFSMs), followed by a second order, where researchers’ interpretations actively linked patterns of meaning with conceptual interpretations. First, we familiarised ourselves with the data through initial readings and revising of the transcripts, which were read, re-read and revised regarding ‘inaudibles’ and inaccuracies. Second, the fieldwork team (NC, MH, MOD and PS) made notes and started generating initial codes for building the skeleton of the coding frame and then collectively coded the dataset in NVivo14. For instance, draft codes included ‘food preferences’ and ‘increased knowledge’. Third, we searched for themes by examining how codes clustered into potential patterns relating to children’s preferences, children’s learning from interventions, families’ eating practices and healthy food. Fourth, we reviewed potential themes to check for coherence and reallocated or discarded them depending on coded extracts and how they were situated across the dataset. This helped to ensure the analytical narrative reflected the meanings voiced across participants’ extracts. Fifth, we defined and named themes through ongoing refinement in analytical panels and in communicative validation exercises (see below), determining each theme’s organising concept. The codes described above were refined into Theme 2: ‘Encouraging collective agency through food engagement’, reflecting a process through which food education and engagement activities enabled shared agency in children, families, and schools. Finally, we drafted the manuscript by selecting illustrative pseudonymised quotes grounding the findings, linking themes to where they provide an interpretation to the research questions.

#### Analytical panels and reflexivity

We adopted a collaborative approach to analysis [82] through analytical panels, which encourage collective data interpretation [83], as knowledge is understood to be co-constructed and qualitative analysis can be enriched by diverse perspectives [84, 85]. We carried out four analytical panels with community researchers (MOD, PS), whose input strengthened the relevance, transparency, resonance and grounding of findings [85, 86]. These panels functioned as internal rigorous checking mechanisms, facilitating collective reflections based on fieldnotes and consensus-building around reading and discussing the emergent and distinctive themes and codes, ensuring interpretive coherence among the research team. Rather than seeking uniformity or more quantifiable methods of agreement commonly applied in other qualitative approaches [87], our analysis was based in interpretative consensus through the panels [82]. In practice, MOD and PS brought their grounded perspectives based on their local and observational knowledge in schools and of community life. They identified children’s food preferences and eating practices during lunchtime, highlighting portion sizes and food waste, depending on children’s age, backgrounds or dietary needs. Their contribution helped us shape analytical decisions about conceptualising children’s agency and the impact of the food education interventions. They elucidated parental perspectives directly from lived experiences, particularly as PS is a local mother who has experienced the policy and interventions first-hand.

We embraced a flexible and reflexive approach, drawing on the principles of relationality and participation applied to ethnographic methods [54]. As four women researchers from different backgrounds, we were mindful of our positionality and engaged in inclusive practices throughout our interactions with adults and children from diverse cultural backgrounds themselves [88]. Inclusive practices were embedded in the study design and fieldwork, including the use of child-centred, creative methods, being supportive to participants for whom English was not their first language with peer translators; and continuous attention to power dynamics in our research encounters in the field. This was also facilitated by working closely with schools to include children with special needs and adapting our materials and tools to be accessible. We critically reflected on our positionality in our ethnographic diaries [54, 56] which were regularly discussed during fieldwork and analytical panels.

### Findings

#### Sample profile

The sample reflected the demographic characteristics of our local population, displayed in Table 2:

**Table 2:** Sample demographic profile of six primary schools*, detailing children, parents and school staff interviewed

Demographic characteristics	Categories	Participants (*N*)	Participants (%)
Parents’ age	25–34 years	2	10%
	35–44 years	14	70%
	45–54 years	4	20%
Ethnic background (Parents)	Bangladeshi	7	35%
	Black African	5	25%
	Black Caribbean	1	5%
	White British	2	10%
	White Other	2	10%
	Mixed	2	10%
	Turkish	1	5%
Parents’ employment	Part-time employed	7	35%
	Homemaker	6	30%
	Unemployed	3	15%
	Full-time employed	2	10%
	Student	2	10%
Children by school year	Year 2	8	18.6
	Year 3	4	9.3
	Year 4	5	11.6
	Year 5	14	32.6
	Year 6	12	27.9
Staff roles	5 Headteachers, 3 Senior Management, 1 Teacher, 6 TAs/Supervisors, 1 Kitchen staff	16	N/A

*Participating schools had fewer than 200 to over 400 pupils regarding school size and 50%–69% of pupils eligible for free school meals, indicating high socioeconomic disadvantage.

Thematic analysis unpacked four key themes exploring the role of school food-based initiatives such as UFSM and the FFiS programme to promote equity, foster agency, support cultural identity, and engage families, while recognising capabilities and limitations. They include: (1) UFSM accessibility with a whole-school approach: a tool for health equity; (2) Encouraging agency through food engagement; (3) Schools as sites of sociocultural negotiation: food anchoring culture and identity; and (4) Capabilities, institutional responsibility and limits.

### Theme one: UFSM accessibility with a whole-school approach: a tool for health equity

The UFSM offer was welcomed by all participants and seen as crucial to help mitigate food insecurity and promote equity by ensuring that every child, regardless of socioeconomic background, receives a nutritious meal at school. Given that UFSMs have been offered for more than a decade in Tower Hamlets’ primary schools, they were perceived as central to support children and families in their everyday functioning and promote children’s healthier eating. Many parents saw this long-standing provision as a normal part of school life, often unaware that it is not universally available elsewhere. As one school staff member identified:

*“Funding [of UFSMs] is a really good result for children*,* purely because everyone will get a hot meal every day in school. […] because many children are from a deprived background and this is the best meal they have for the day so […] you feel good knowing they’re going to be fed.” (Staff member)*.

All parents appreciated the financial relief UFSMs provided, especially given the cost-of-living crisis. For many, UFSMs have become a reliable daily food source to count on. One mother expressed:

*“There was a gap between the parents like myself*,* I had kids in school and having to pay for all of them for their dinner*,* but didn’t earn enough money really to pay for their dinner*,* and that was a struggle […]. That was very*,* very hard […] Now that everybody gets it free*,* that means that every child gets a chance to have a possible hot dinner or food.” (Mother of 4)*.

#### Breakfast clubs: accessibility and wellbeing through play

Beyond UFSMs, whole-school approaches to food are evident in breakfast clubs. Our observations captured how breakfast clubs expand food accessibility by offering children an additional meal in a supportive, social space. In one school, they provided a holistic environment, equipped with sensory play installations and a relaxed atmosphere with the radio playing in the background. Caring staff fostered social interaction and supported children with mental health or additional needs, as exemplified by the following excerpt from this fieldnote (Table 3):

**Table 3:** Ethnographic reflections 1 from fieldwork diaries on observations of breakfast clubs

“*Around 8:30 am, children move to the adjacent playground, equipped with ludic and sensorial tools with wind music installations and painted roads, all under canopies. I feel how this inviting environment is conducive to play, promoting sensory engagement, including children requiring mental health support.” (Reflexive fieldnotes 1, breakfast clubs)*	

The breakfast clubs were perceived as key in helping parents who had additional health and economic support needs, as this mother explained:

*“I love coming to breakfast club with my little one*,* because I’ve got MS and I do physio. So in the morning it’s really good for me to bring my little one just to drop her. It’s really good. It’s a lifeline for me. Because in the morning I’m terrible and it’s better for me to come and just have at least 10 minutes to myself to get my stuff*,* put my leg brace on […] and then I have to go to my leg hospital. So*,* yeah*,* it’s amazing*,* breakfast club*,* cause other schools I used to pay. Here*,* it’s free. Some schools you pay breakfast club which is not good nowadays.” (Mother of 4)*.

Some mothers discussed how breakfast clubs gave children an opportunity to do additional activities and socialise with their friends, as the following excerpts demonstrate:

*“When I was working*,* [child] used to come breakfast club because I’d have to drop him off early and go. And he always liked breakfast club. I think*,* because also they do activities as well*,* so they’re having their breakfast and then when they’re done with their breakfast*,* they can move on to the different activities on the tables*,* yeah.” (Mother of 2)*.

*“I said why you don’t like Mummy’s breakfast*,* but you like the school breakfast? ‘I see my friends’. And what else? What’s so special about the school? So I can do it? And she’s*,* and she said ‘playing the activity’.” (Mother of 4)*.

#### Institutional responsibility and food justice

Schools play a vital role in promoting food justice and healthy food provision but they are also finding their resources stretched in the context of food insecurity.

*“I’m under a huge amount of pressure at the moment because of budget*,* so I’m spread very thin. I work four days or 4 1/2 days and we’re just managing […] We’ve got very limited lunchtime ambassadors […] we’re all doing too much and have too much to do” (Staff member).*

They find themselves balancing new roles and responsibilities for supporting families with systemic issues such as food insecurity, including providing food parcels, linking in with food banks, breakfast initiatives, charities and supermarkets.

*“I have had more requests from mums*,* particularly single mums*,* for me to give them a job. [.]. I’ve never been asked for that before. They’re desperate. They can’t make the money stretch. Could I find a job for them? Could I find something around the school? Not*,* not above board” (Staff member)*.

These additional needs present difficulties in achieving healthy eating, as one staff member reflected:

*“There’s so much food poverty. Children will say they don’t have the fruit at home because the parents are trying to focus on a meal that’s going to fill them up. So*,* they’re having to make choices about what are they buying. With deprived families: are they going to buy that bag of pasta or some fruit? By us offering that fruit*,* that’s taken some pressure off the family.” (Staff member)*.

*“It’s [cost of living] impacting our family […] I’m sure it’s impacting other families in the school […] they may be eating more junk food. Because it’s cheaper to buy processed pizza that is already made.” (Mother of 2)*.

Pressures to meet social needs can be overwhelming. Meeting them depends on the commitment and engagement of staff in implementing additional support systems and not all schools and staff can achieve them.

In summary, factors explored in this section uphold the programme’s potential to improve equitable healthy eating, addressing research question 1 (RQ1), where parents and staff felt that universal provision responded to the particular economic needs of the community. Thus, healthy eating was seen as more equitable as all children were on a level field. The healthy whole-school approach to food was expanded through the implementation of breakfast clubs that catered to children’s needs. However, food insecurity factors also created challenges for the programme as schools struggled with stretched resources.

### Theme two: encouraging collective agency through food engagement

Although systemic food insecurity is difficult for schools and the FFiS programme to tackle alone, impactful changes were found within their remit.

#### Child agency in food education

The FFiS initiative enabled a positive food environment by actively engaging children in interactive, innovative and educational activities through child-centred interventions breaking their daily school routines. The TastEd (Taste Education) sessions were designed to expose children to a variety of fruit and vegetables through embodied, sensorial experiences, broadening their exposure to unfamiliar foods, by creating metaphors, for example:

*“The apple sounded like the floor was rumbling.” (Girl*,* Year 3)*.

The playful and in-depth sensory engagement enabled children to apply learning to their lived experience. Staff highlighted how these activities supported the curriculum, particularly by encouraging literacy. TastEd was also seen as a vehicle for teaching consent and role modelling, encouraging children to make informed choices about the foods they encounter:

*“Consent can mean that you’re empowered to say no*,* you’re empowered not to like something […] [TastEd] is a real good vehicle for consent.” (Staff member)*.

Similarly, a Cook-a-thon TV style competition was enthusiastically received by pupils, generating excitement and engagement with healthy food preparation. The competitive nature of the event, coupled with a hands-on cooking experience, helped children share and increase their knowledge of nutrition, food groups and cooking. Children told us the following:

*“The Cook-a-thon was epic!” (Boy*,* Year 4)*.

*“We learned a lot from the Cook-a-thon!” (Girl*,* Year 5)*.

A member of staff reflected on the impact of the Cook-a-thon on students:

*“I was really impressed by the level of knowledge that our children had as well. So they were very knowledgeable about food groups*,* about nutrients*,* about where food came from.” (Staff member)*.

These activities enabled collective agency by encouraging children to become familiar with healthy foods. One Year 5 girl voiced this:

*“I can get used to healthy foods if I eat them more and I can get more healthy and start to like it a little bit.” (Girl*,* Year 5)*.

Importantly, the increased confidence children expressed extended beyond the school setting towards family agency. Healthier food practices in school extended beyond the boundaries of the school and into the home environment. One mother reflected on the impact of these activities on her child and the wider family:

*“My daughter*,* she do something in the school*,* […] easy*,* nice and healthy. She get more confidence to come home and she say: ‘I want to be healthy. I want to do as we done in the school.’ […] she encouraging me also to be with her to teach her something new about healthy food.” (Mother of 2)*.

#### A community approach to healthy food

Parents are key social actors in the school food environment. Efforts to engage parents and families in creating a healthy school-community food culture were recognised by staff and by parents themselves. One example illustrates how targeted engagement through the programme led to tangible child health improvements. Through one-to-one support, a mother who struggled to manage her child’s behaviour changed her family’s eating practices and the boy achieved a healthier weight. A staff member reflected on the outcome:

*“One mother changed her son’s diet and did put the crisps away and the biscuits away and stopped buying them. And he lost a lot of weight and he’s much healthier weight. […] he was […] obese when he came to us.” (Staff member)*.

#### Parent tasters: dialoguing around school food

The programme also created spaces for dialogue around food with parent tasters, bridging parents’ knowledge of school food with home eating practices. These sessions provided a platform for parents to experience school meals firsthand and share experiential understandings of school food with their children. This led to more positive perceptions of school food among parents.

*“M1: Today’s [Parent taster] food I liked*,* colourful as well.*

*M3: […] Today was beautiful. [.] I love the coleslaw.*

[All talk at once]: The salad was the highlight. Yeah, yeah. The salad, yeah!

M3: It was eye catching.” (Interview, 3 mothers)

In this second theme, we have explored how different elements of the FFiS programme were received well by staff, parents and children (RQ2). Children were perceived to increase in knowledge and confidence with regards to healthy food, which they shared with parents at home (RQ3).

### Theme 3: schools as sites of sociocultural negotiation: food anchoring culture and identity

Food is deeply embedded in social identities, and this was evident in the diverse borough of Tower Hamlets, where we observed that food can serve as a unifier and a source of tension. Some parents, children and staff highlighted that culturally diverse foods were, in practice, not always catered for. School menus, designed with public health and food standard guidelines in mind, did not align with familiar preparation methods in the home. One staff member recalled a parent telling them:

*“You’re changing the food so much you can’t even call it that anymore. There’s no way*,* we don’t want baked samosas. They’re not samosas!” (Staff member relaying a parent comment)*.

The gap between what local families symbolically associated with authentic dishes and health-conscious meals highlights the barriers faced:

*“Because we are from Asian background*,* when we cook*,* we use lots of spices*,* lots of salt*,* lots of oily thing. So the children are used to having food*,* whose got spices. […] they [school] have different ingredients and different measurement to use. So when they have the school meal*,* no*,* it’s not nice. It’s bland!” (Mother of 3).*

As such, school celebrations can make balancing nutritional goals with embedded food representations and practices difficult. However, reported positive changes over the course of the programme were led by children’s knowledge gains and adherence to school food standards, gaining parental acceptance:

*“My son has his birthday in two weeks’ time*,* he said no chocolate*,* nothing that contains sugar*,* not this not that and he’s only 5! […] He himself knows which one is healthy*,* which one is not. So I think in this way this school’s done very well.” (Mother of 4)*.

#### Balancing food preferences with health needs: a nuanced tension

Similar issues were raised by children regarding the perceived quality, portion sizes, and lack of culturally appropriate food. This was prevalent in mini-groups with children in Years 5 and 6 (10–11 years), who particularly raised issues with portion sizes:

*“Boy 1: I think they should give us more*,* big portions. Yeah*,* big portions. Because we’re older yeah*,* we need more energy.*

*Girl 2: Yeah*,* when I go home*,* I’m always hungry. I always have lunch when I go home.” (Mixed gender group*,* Year 5)*.

Others expressed dissatisfaction with limited menu variety but days like “Fishy Fridays” were particularly anticipated, while others voiced frustration with menu changes or vegetarian meals as main options:

*“Sometimes we don’t like the food […] There’s no other option*,* and they make way too much vegetarian food.” (Boy*,* Year 5)*.

This reveals a recurring nuanced tension between cultural and familial food preferences, often tied to notions of comfort and familiarity, with the programme’s public health responsibility to promote healthier eating and avoid food waste. However, findings also suggest that this dynamic was more complex. Despite preferences for home meals, some children acknowledged liking the different menu options offered by their school, particularly with meals they did not consume at home, such as chicken roast with gravy, broccoli, mashed potato and wedgies.

*“Boy*,* Year 6: I like any lunch day [when] they have like meat with wedgies. The wedgies are nice.*

*Girl*,* Year 5: I like broccoli*,* it’s my favourite.*

Boy, Year 6: And the roast with gravy.” (Mixed gender group, Years 5 and 6)

Children also displayed openness to trying new foods when they were introduced in an engaging environment by staff. In one school, visual tools and active discussions in the lunch hall helped them learn about unfamiliar foods:

*“Some children look at the food and because they’re unfamiliar*,* they’re scared to try new foods […]. We have an overhead projector in our hall*,* the children are shown visual pictures of the new food every day. […] we have the picture to encourage them*,* we have a different kind of chit chat topic […] explain to them that this is about you trying new foods and you have the enthusiasm.” (Staff member)*.

Despite some varied acceptance of school meals and openness to try healthy foods in an inviting environment, general challenges UFSM programmes continue to face reflect a misalignment between what children *want*,* find familiar* and what they *need* to be healthy. This was clearly outlined by staff:

*“They want children to have a nutritious meal every day and be healthy and be ready to learn [.]. But they’re also wanting children to try new things and […] eat more healthy*,* […] they don’t quite fit together sometimes because if you want children to eat*,* you want to give them something that they enjoy*,* that they recognize. But it has to be healthy.” (Staff member)*.

This nuanced tension was apparent during our observations and when meals were shared with children in school lunch halls, where some children often resisted trying particular, unfamiliar foods or finishing their plates, resulting in food waste (Table 4).

**Table 4:** Ethnographic reflections 2 from fieldwork diaries on sharing lunches with children

“*Sitting with children sharing lunches, it became apparent how their food preferences were enacted. On less popular lunch menu days, children would be sitting in front of me and discreetly drop food on the floor. This act, at times hidden and deliberate, seemed like their own expression of agency. Conversations with lunch meal supervisors revealed that finishing the plate was a daily negotiating exchange for children to receive a healthy pudding or be allowed outside to play. Dropping food became a quiet yet meaningful form of resistance—a way for children to navigate adult expectations and assert their agency within the structured school food environment” (Reflexive fieldnotes 2, sharing school lunches).*	

Food waste is a challenge all schools face and it is particularly demoralising for kitchen staff, as one expressed:

*“It’s horrible to think I’m cooking for the bin.” (Staff member)*.

#### Improvements in school food quality

Despite the tension and food waste issues, school menus improved over the course of the seven-month data collection period. Tastier food options were positively received, addressing earlier issues.

*“Now we’ve jiggled the menu about they’re [pupils] a lot more happier with the options that they’ve got*,* whereas before it’s like you could see them coming out and their face says it all. […] But now at least I know that there’s something that they’re going to like […]. Year sixes*,* they’re liking the menu.” (Staff member)*.

These changes were noted by our team directly when we shared meals with children, reflecting the programme’s responsiveness through joint working among the lead chef, the centralised catering service, kitchen and teaching staff.

In terms of acceptability of the programme with participants, we have highlighted some of the challenges and how this were overcome (RQ2), in particular regarding changes to the school menu and responding to diverse cultural food preferences.

### Theme 4: Capabilities, institutional responsibility and limits

Despite recognised improvements, schools were at different points in the trajectories of implementing the programme. Various levels of involvement from kitchen staff across schools were found and some tensions were reported.

*“[regarding feedback session] there’s a bit of a disconnect and I think […] kitchen staff are a little bit confused because they feel criticised again.” (Staff member)*.

Introducing menu upgrades, based on survey consultation feedback, added pressure on kitchen teams, contributing to tensions. Feedback came from multiple perspectives—students, parents and staff—requiring catering services and kitchen staff to adapt. School staff acknowledged the integral role of kitchen teams, but also highlighted issues where more training was required, recognising that a joined-up approach involving them was key to mitigate these challenges. A staff member highlighted:

*“You need to get your kitchen staff on board because it is more work for them.” (Staff member)*.

#### Peer knowledge sharing

Schools also learned from each other and were scaffolded by the council through peer knowledge sharing, which occurred in different ways across the programme. The council supported these collaborative efforts by facilitating opportunities through a wider working group, particularly with the initial participating schools. This wider peer involvement provided a valuable platform for schools to learn from each other, building collective agency to enable change:

*“I’ve visited other schools and looked at how they did lunch*,* and have learned from ones that are doing it well. […] I’ve said […] we really want to stop giving children puddings because that’s not healthy and it harks back to our days at school […]*,* chocolate pudding in custard and all that sort of stuff. And then I talked to [staff] who really pushes for healthier foods in schools*,* so I’m thinking ah*,* it’s possible!” (Staff member)*.

#### Not all interventions ‘fit’: family-style dining

Another intervention we observed throughout the programme was family-style dining, which involves children sitting together and serving themselves from shared dishes, following a home-like dining experience. Most staff agreed in principle with this intervention, discussing its social value and potential for food waste reduction. Those that implemented family-style dining, at varying stages, discussed how it promotes agency, exposes children to diverse foods, and may enhance social skills and development related to communal eating:

*“[…] the dinner table is a place for developing social skills*,* language*,* sharing*,* […] the mealtime is a time where children stop. Talk. They’re social. They get on with each other. […] that’s what we’re here to do as much as the academic learning*,* is teaching social skills.” (Staff member)”*.

Some young children in the study arrived at schools with less experience of using cutlery; this is particularly relevant in Tower Hamlets, where hand feeding is common amongst Bangladeshi families. Family-style dining facilitated children to learn to use cutlery in institutional and dining spaces, like schools, where this is expected.

*“He [son] still struggles*,* how to hold a spoon. So when he’s holding a spoon*,* half of the food has gone back on the plate or on the floor and tiny bit in his mouth.” (Mother of 2)*.

However, differences in school infrastructure, budget constraints, staff capacity, resources and involvement indicated that resource-intense interventions such as this one require significant support for staff and might not work for all schools.

*“They came up with many ideas*,* but we will only implement what works for us. […] some schools extend the hours*,* which we can’t do*,* […] there’s some staff to sit with them*,* which we can’t do*,* […]*,* you can’t change the timing. But we will take on board what works for our school.” (Staff member)*.

Staff sharing meals with students was encouraged in some schools but not all teachers participated. Some teachers expressed a need for breaks, as these demands can be difficult for overworked staff, particularly given the added pressures they face in their shifting roles and responsibilities, such as having to support parents and families in the current cost of living crisis with rising levels of food insecurity.

This final theme connects with RQ2 where we reflect on the delivery of the programme, and the feasibility of implementing it in practice, highlighting the institutional conditions within which equitable whole-school approaches to food can be realised (RQ1).

## Discussion

We have shown that UFSMs and whole-school food initiatives like the FFiS programme can help mitigate the inequalities exacerbated by food insecurity in urban dense, culturally rich areas such as the London Borough of Tower Hamlets. These initiatives contribute to the broader *SDG 2* mission to end hunger and achieve food security. Our findings suggest that UFSMs is a much-needed policy in England, particularly in areas of high deprivation, resonating with international research on UFSMs and school meal programmes addressing food insecurity and child poverty [89–92]. Public health experts, researchers, and free school meal activists [34, 93, 94] have called for an expansion of UFSMs nationwide to address widening child health inequalities. Our findings align with those calls but also recognise the tensions inherent in school food provision between meeting conflicting dietary preferences and needs, as well as in tailoring food provision for diverse local populations. Factors such as portion sizes for older year groups, food quality, and access to culturally appropriate meals, must be understood through the symbolic meaning and cultural significance that food holds for diverse communities, requiring a sensitive and tailored approach to ensure that healthy eating practices align with diverse needs. This resonates with research drawing on the importance of providing culturally appropriate food options and fostering an inclusive dining social environment to improve meal uptake and participation in school meal programmes [28, 42, 61]. This further aligns with the conceptualisation of school meals as a form of “culinary capital,” where food is deeply connected to social resources, practices and cultural identities [61].

Our research contributes to the evidence base on whole-school approaches to food, offering insights that can inform future policies to improve school food programmes. Breakfast clubs were perceived as providing an important meal in addition to UFSMs, enabling social interaction and supporting children and parents with additional needs. This is consistent with other studies indicating that breakfast clubs not only provide nutritional benefits but also foster social relationships [95]. This dual function highlights the importance of addressing both individual nutritional intake and the broader food environment [39, 96]. Whole-school approaches to food, enhanced by interactive and hands-on learning, enable children to make healthier food choices in an encouraging environment, reinforcing the development of positive food behaviours from an early age [97]. When implemented in the ways in which we have shown, this approach to school meal programmes can further benefit children who are experiencing food insecurity.

### Impact of child-centred and relational approaches

The FFiS initiative fostered positive relational dynamics and improved school food knowledge and culture by delivering engaging and inclusive interventions. All the social actors involved in the school community reported high levels of satisfaction with these activities, particularly the child-centred interventions. Initiatives such as TastEd and Cook-a-thon can help to bridge health education with everyday school life and include playfulness, enthusiasm, and fun embedded in the learning process to further enhance social development and wellbeing [97]. Indeed, firsthand involvement of children - as well as parents - not only informed parents about their children’s meals but also created opportunities for meaningful discussions about food. Beyond providing access to nutritious meals, teaching children how to make informed healthier food choices is crucial in shaping long-term health outcomes, by fostering a culture of health through engaging practices, aligned with whole-school approaches to food [39, 57, 96]. The influence of food education on children’s food choices and behaviours is supported by research that demonstrates the value of interactive, hands-on learning, exposure and involvement in shaping attitudes towards healthy eating [98–101]. Similar peer-led and beneficiary initiatives have been found to foster increased confidence and a sense of collective agency, where schools and communities can advocate for policies that better reflect their needs [39, 94].

### Challenges and systemic barriers

However, school food provision alone is not enough to address the complex health inequalities faced by children in economically deprived areas. Social determinants like poverty, educational opportunities, and household conditions play a profound role in shaping children’s health outcomes [102]. These factors create additional challenges for food provisioning in schools, which remain multifaceted. Hawkins and Rundle (2023) argue that systemic barriers—particularly power imbalances among schools, public health advocates and the food industry—bring significant difficulties to the implementation of UFSMs and school meal programmes. This perspective aligns with our findings, but we did not examine larger systemic factors such as national food policies, commercial determinants of health and the influence of the food industry [103], or broader socioeconomic structures driving UK health inequalities [102]. Transforming food systems at the macro-level, through public procurement reform and supporting sustainable local food production, can help bridge these gaps and ensure that school meal programmes are better aligned with health, thereby meeting the aims of *SDG 2: Zero Hunger* and related SDGs, including, *SDG 3: Good Health and Well-being; SDG 12: Responsible Consumption and Production*; and *SDG 10: Reduced Inequalities*. The *FixOurFood* project, part of a wider funded UK initiative, focuses on transforming our food systems by promoting sustainable, local supply chains and addressing inequalities in food access [104, 105]. Future London-based school food provisioning projects like FFiS, would benefit from transferring learning from similar initiatives to improve their food system transformations.

### Localised delivery and collective agency

Despite challenges, our study found that localised implementation strategies are key. Tailoring school food programmes to meet the capabilities, capacity and resources each school has to be sensitive to local needs, means being reflexive to understand unique contexts. For instance, efforts can focus on improving relational dynamics and communication channels amongst kitchen and school meal supervisors to enable better integration of school lunch delivery. Involving kitchen and school lunch staff has also been found to be integral for guiding children towards making healthier choices, by introducing food options and engaging with children during meal times [43]. It could help support meal supervisor staff to navigate the difficult tension between ensuring children experiencing food insecurity eat enough whilst managing childhood excess weight, particularly as portion sizes tended to vary across the schools. Improving lunch routines entails recognising variations in leadership and school structures and, importantly, that not all interventions fit across all contexts. Implementing initiatives like family-style dining, which were seen as resource-intensive, require additional support and resources. This is important for adherence, increased uptake and sustainability. The efforts that schools and councils must make to try and address the broader and multiple barriers they face to implement effective, equitable school food programmes must be recognised, precisely because the macro-level pressures on schools and local authorities are huge [60]. As such, looking at the shorter-term, we call for continuous review and improvement of the school food menu offer and wider adoption of whole-school approaches to food so that more children are supported to develop healthier eating practices. Policymakers and priority setting must result in sufficient resources allocated to these initiatives, ensuring that schools have the financial resources, infrastructure and staff training necessary to work towards the goal of enabling an equitable and inclusive food environment that meets the diverse needs of children, regardless of their family’s socioeconomic conditions.

Ultimately, applying a collective agency model to food programmes [106] can support schools and local councils to take a more active role in working with the UK centralised education system to embed school food literacy and practice into the curriculum. Extending collaboration by linking with the third sector and private food suppliers can also help address some immediate food insecurity faced by local children and families, as our study revealed. A holistic approach to health is needed, through wider linkages to support parents in seeking job opportunities or training schemes that could reduce food insecurity and financial pressure on families. However, to ensure the sustainability and broader health impact of these programmes, we need to reach national and international governments to help address the macro-level factors influencing food systems and policy, as these determine the resources, regulations and opportunities available for schools to succeed [60, 97, 103, 104].

### Strengths and limitations

Our approach, based on a focused ethnography, was inclusive of the school community, including children, parents and school staff. By exploring interconnected material, relational, and symbolic factors [63, 64], with researchers attuned to the contextual significance of the field [78], our findings can contribute to local policy by highlighting how complex and intertwined food environments shape health eating practices. The study was innovative by directly including children’s knowledge of and experience with school food. Parents and school staff cannot provide the same sensorial perspective as children, who have school meals daily. As direct beneficiaries of UFSMs, children actively informed council decision-makers about their food preferences and practices through their participation in the study. Our methods aligned with established participatory principles [66], by engaging with the local community and working with community researchers, as well as using visual narratives, playful methods, and sharing meals with children. This reflects Earl’s (2022) ethnographies of eating and sensory ethnography when exploring school food provisioning. Incorporating both observational and interactive methods in the focused ethnography allowed for a rich, in-depth exploration of the lived experiences of children, parents and staff.

Given the timeline of the FFiS, our sample concentrated on schools which were early adopters of the programme and therefore more likely to be invested in whole-school approaches to food. We were unable to collect individual details from children regarding ethnic backgrounds or SEND (Special Educational Needs and Disabilities) status, though we asked schools to be inclusive when inviting participants to take part. Recruiting via schools poses challenges and the process to enable children’s involvement in research is complex [107]. Capacity, time and other pressures on school staff may have limited their support for research, which may have influenced the sample. However, all the participating schools were invaluable in enabling access and fieldwork. While 34.6% of Tower Hamlets’ overall population identifies as Bangladeshi, the proportion is higher among school-aged children, where 61% of Year 6 pupils were from Bangladeshi backgrounds in the 2023–24 school year [108]. Our parent sample reflects the borough’s broader demographic profile, with 7 out of 20 (35%) parents self-identifying as Bangladeshi [109]. Most parent participants were mothers (*n* = 19), with only one father. Despite working with schools to encourage fathers to participate, the gendered dynamics of childcare responsibilities, present to a large extent in the UK, became evident [110, 111]. A final limitation of this study lies in its specific setting in Tower Hamlets, where UFSMs have been delivered in primary schools for over a decade [35, 112]. This long-standing provision means that UFSMs were largely taken-for-granted by schools and families, which differs from areas where UFSMs have been recently introduced (e.g., across London’s primary schools in 2023), partially implemented, or not offered. As such, our findings may not be transferable to places with different school food provisioning models in the UK or internationally.

## Conclusions

The FIGS I study contributes qualitative insights to the growing body of evidence supporting school-based interventions as a crucial platform to reduce health inequalities and promote lifelong healthy eating habits [22]. The UNCRC principles on the rights of the child highlight the importance of involving children in decisions that impact their lives. By applying these principles to food-related practices and engaging parents, we can strengthen participation and connections between the two most important environments in a child’s life: the home and school.

The relevance and timeliness of these findings are clear. They can inform the ongoing implementation of the FFiS initiative across Tower Hamlets’ primary schools, the extension of the UFSM scheme to all primary school children in London and support a wider implementation of UFSMs nationally. Finally, our study serves as an exemplar for collaboration and partnership building between schools, local government, academics and local communities. By collectively generating knowledge to directly inform policies, we can be more responsive to the lived realities and perspectives of local children and families, with more contextually grounded and culturally relevant interventions. This partnership approach, in turn, helps drive the global health commitment to meet *SDG 2: Zero Hunger* and ensure equitable access to healthy and nutritious food for all. 

## Supplementary Information

Additional file 1.

## Data Availability

The qualitative data that support this study’s findings are available from the corresponding author, NC, upon reasonable request. Access to the data is restricted due to privacy and ethical considerations per our institutional ethical approval, as participants were assured confidentiality and anonymity during data collection. Therefore, the data are not publicly archived to protect the ethical agreements and privacy of the participants. Requests for access will be assessed on a case-by-case basis to ensure compliance with ethical standards. All the material is owned by the authors and/or no permissions are required.

## Abbreviations

DBS: Disclosure and Barring Service
FFiS: Fantastic Food in Schools
FSMs: Free school meals
UFSM: Universal free school meals
UK: United Kingdom
WHO: World Health Organization
