# Supplementary material for: Food Improvement Goals in Schools (FIGS I): a qualitative evaluation of a whole-school approach to healthy food provision in primary schools
Source: BMC Public Health. 2026 Apr 22;26:1798. doi: 10.1186/s12889-026-27503-0 (PMC13235117; doi:10.1186/s12889-026-27503-0)
Supplement: Supplementary file 1 — Additional file 1. [file 12889_2026_27503_MOESM1_ESM.docx]

Additional file 1:

Data instruments pack

This document provides the data instrument tools to guide the fieldwork. There are a range of participants and – although many areas of questioning are common – 4 Topic Guides and 1 observation template have been produced and outlined below.

*Note*: The topic guides are not meant to be used as a ‘question-by-question checklist’; they are flexible to enable participants to discuss what matters mostly to them in relation to the broader topics we are exploring. These will also continue to be developed iteratively, as the fieldwork progresses.

| **Participant Type** | **Instrument** |
| --- | --- |
| Teaching staff - Providers | 1 |
| Kitchen staff - Providers | 2 |
| Parents/carers - Beneficiaries | 3 |
| Primary age school children – Direct beneficiaries | 4 |
| Observation template for fieldnotes | 5 |

Guide for school staff (Topic guide 1)

| **Researcher - Please provide the following**:   - Introduce the FIGS Study. The interview is divided into two parts: 1) explore how food is consumed at the school: free school meals (FSMs), packed lunches, fruit and vegetable schemes, breakfast and afterschool clubs and 2) gather their views and experience of the Fantastic Food in Schools (FFiS) Programme. - Interviews will vary because of differing degrees of involvement staff will have with school food provision and the FFiS. - Emphasise that there is no right/wrong answer, this is a discussion to capture their views and experiences of school food provision and the FFiS. - **Informed consent**: Emphasise all information given will be treated in complete confidence – no individual will be identified in any reporting and their views will remain confidential, all transcriptions and extracts will use pseudonyms.   - Permission to record interview (audio) - **Duration:** ~1 hour - **Researcher:** The order of questions can change as the interview moves on. It is key to study the guide and be clear about questions in each section. - **Probing questions**: Probing questions follow from general, open questions, and then can support you to gather more detail and in case the interview loses ‘flow’. |
| --- |

**Part I: Description of school, role and food provision**

**School: pupils and staff**

- Please describe the primary school and their role within it.
- How many pupils does your school have? [Probe: size (1-3 entry form), type, pupils’ demographic profile, teaching staff].

**School food provision: Free School Meals (FSMs) and catering**

- Please describe your school’s food provision (i.e., school dinners and FSMs, breakfast/afterschool clubs, fruit and vegetable schemes, etc).
- Roughly, what’s the uptake of FSMs?
- Roughly, how many of your pupils are eligible for FSMs? And how many of those take up FSMs?
- How do pupils sign up for school dinners on a day-to-day? [Probe: Per term/flexible?]
- How do you/school communicate with others in the school community about food offers (FSMs/holiday school vouchers/breakfast-afterschool clubs)? What messages do you use (if any)?
- Who are your food providers? (i.e., contract services/in-house, etc)? How does provision work in the day-to-day? How long have they been providing meals to the school?
- What is the school menu on offer?
- How do teaching staff work around food provision?

**Teaching staff’s meta-perspectives on school meals and packed lunches**

- Can you describe to me a typical school lunch. What happened [yesterday/last school day] during this time in your dining hall? How do children use the space to eat their meals? How do they interact?
- What do you think your pupils think of school dinners/packed lunches? Does this vary by year? By pupil characteristics? (i.e., dietary requirements)
- Do you have a sense of why pupils have/don’t have FSMs? Why they like/dislike them?
- What do you think are the reasons why parents/carers prefer one type of lunch (packed lunch vs FSMs) for their children?
- What do your staff think about these offers? Does this vary?
- **UK cost-of-living crisis**: are there any difficulties your pupils and their families might be going through? Do you know if this affecting what they can/not eat?
- Can you remember a time when you felt proud about the way food is provided at your school?
- Can you remember a time when you felt like the school could do a better job at providing food for the pupils?

**Part II: Fantastic Food in Schools Programme (FFiS)**

**Views and overall experience**

- Let’s talk about the FFiS. Tell me how it has been implemented at your school.
- What are the interventions/activities you’re running as part of it? If none, why is that? [If none, reframe questions in terms of planning]
- How are they delivered? [Probe: additional food offers, connected to the curriculum, classroom activity/lessons]? External providers?
- What has worked well in the programme? Is it been delivered as planned?
- Is there a school food culture been implemented? Which staff are involved in the FFiS?
- How does this work with Tower Hamlets council?

**Implementation: Resources, training, capacity and support**

- What resources have been/are used in the FFiS [activities mentioned]?
- What do you think of these? Are they suitable for your school’s needs/ community?
- Have you/colleagues received any support to implement these? If yes, from whom? And how? If not, why do you think that is?
- Did [mentioned above] involve any training? If yes, what sort of training? [Probe: who delivered it, where, how?]
- What did you/staff think about this? Helpful/not?
- What additional support (if any) would you like to receive from the council?

**Challenges to implementation**

- What challenges (if any) have you faced/facing in the programme? [Explore: logistics, planning, time constraints, capacity, resources and prioritisation]
- Have there been any issues in incorporating FFiS initiatives into the school?

**Perceived impact of FFiS**

- What do you think has worked well for your school?
- How has the programme been received by staff?
- Has the FFiS influence healthy eating in your school? What about wider outcomes?
- Have these interventions contributed to expected outcomes?

***Community beneficiaries: Pupils and parents/carers***

- What are pupils’ involvement (if any) in [initiatives discussed already]?
- What have pupils thought about these activities?
- Have [activities/intervention(s) mentioned] made a difference in pupils? [Probe: knowledge on healthy foods, attitudes towards healthy eating, eating practices?]
- Are they engaged with your school food culture? [Probe if influencing]
- Have you seen any other changes in pupils?
- What involvement (if any) do parents/carers have? If they are not involved, why do you think that is? How do you think they could be more involved?

**Part III: Recommendations and looking ahead**

- What’s the best way forward to improve any of these initiatives (if not already discussed)? [Probe: main issues raised throughout the interview].
- Are there lessons/shared learning from your experience that can be used to inform the FFiS roll-out?
- What could help to overcome these?
- What elements of the FFiS would you want to maintain (if any) at your school?
- What might make these more sustainable?
- If you had an unlimited budget, what changes would you make to the way your school provides food?
- Are there any future plans for other healthy food provision/nutrition programmes to be implemented at the school?

**Part IV: Close**

- Do you have anything else you would like to add that we haven’t covered?

***Closing comments and provide a summary of the study***

| *We thank you for your interest and time in taking part if this study to help us better understand how children eat different types of food in their primary schools in Tower Hamlets. This is part of a programme where Tower Hamlets council is helping primary schools to improve their meals and support children to eat healthier. It is part of ActEarly, a wider initiative where universities, councils and communities come together to help improve children’s health.* |
| --- |

***Do you have any questions you would like to ask us about the discussion we just had or the study?***

***Remind them they can contact us at any time. Thank participant and discuss incentive for the school.***

Guide for Kitchen Staff (Topic guide 2)

| **Researcher - Please provide the following:**   - Introduce the FIGS Study. The interview is divided into two parts: 1) explore how food is consumed at the school: free school meals (FSMs), packed lunches, fruit and vegetable schemes, breakfast and afterschool clubs and 2) gather their views and experience of the Fantastic Food in Schools (FFiS) Programme. - Interviews will vary because of differing degrees of involvement staff will have with school food provision and the FFiS. - Emphasise that there is no right/wrong answer, this is a discussion to capture their views and experiences of school food provision and the FFiS. - **Informed consent**: Emphasise all information given will be treated in complete confidence – no individual will be identified in any reporting and their views will remain confidential, all transcriptions and extracts will use pseudonyms.   - Permission to record interview (audio) - **Duration:** ~1 hour - **Researcher:** The order of questions can change as the interview moves on. It is key to study the guide and be clear about questions in each section. - **Probing questions**: Probing questions follow from general, open questions, and then can support you to gather more detail and in case the interview loses ‘flow’. |
| --- |

**Part I: Description of school, role and food provision**

**School and staffing**

- Please describe the primary school they work for and their role within it as staff.

**School food provision, FSMs and catering**

- Please describe the school’s food provision (i.e., school dinners and FSMs, breakfast/afterschool clubs, fruit and vegetable schemes, etc).
- What are the catering services you provide?
- How do your services work in the day-to-day?
- How long have you been providing meals to the school?
- What is the school dinner menu on offer?
- How do teaching staff work around food provision?
- Roughly, how many pupils take up FSMs?
- How do you/school communicate with others in the school community about food offers (FSMs/holiday school vouchers/breakfast-afterschool clubs)? What messages do you use (if any)?

**Kitchen staff’s meta-perspectives on school meals and packed lunches**

- Can you describe to me a typical school lunch. What happened [yesterday/last school day] during this time in your dining hall? How do children use the space to eat their meals? How do they interact?
- What do you think pupils think of school dinners? Of packed lunches? Does this vary by year? By pupil characteristics? (i.e., dietary requirements)
- Do you have a sense of why pupils have/don’t have FSMs? Why do they like/dislike them?
- What do you think are the reasons why parents/carers prefer one type of lunch (packed lunch vs FSMs) for their children?
- And what do teaching staff think about these offers? Does this vary?
- **UK cost-of-living crisis**: are there any difficulties children and their families might be going through affecting what they eat?
- Can you remember a time when you felt proud about the way food is provided at your school?
- Can you remember a time when you felt like the school could do a better job at providing food for the pupils?
- If you had an unlimited budget, what changes would you make to the way your school provides food?

**Part II: Fantastic Food in Schools Programme (FFiS)**

**Views and overall experience**

- Let’s talk about the FFiS. Please describe your role (if any) in the FFiS.
- What are the interventions/activities you’re running as part it? If none, why is that?
- How are they delivered? [Probe: additional food offers, connected to the curriculum, classroom activity/lessons]? External providers?
- Which staff are involved in the FFiS?
- What has worked well in the programme? Is it been delivered as planned?
- Is there a school food culture been implemented?
- How does this work with Tower Hamlets council?

**Resources, training, capacity and support**

- What resources and activities are used in the FFiS [activities mentioned]?
- What do you think of the resources? Are they suitable for the school’s needs/ community?
- Have you/colleagues received any support to implement these? If yes, from whom? And how? If not, why do think that is?
- Did [mentioned above] involve any training? If yes, what sort of training? [Probe: who delivered it, where, how?]
- What did you/staff think about this? Helpful/not?
- What additional support (if any) would you like to receive from the council?

**Challenges to implementation**

- What challenges (if any) have you faced/facing in the programme? [Explore: logistics, planning, time constraints, capacity, resources and prioritisation]
- Have there been any issues in incorporating FFiS initiatives into the school?

**Perceived impact of FFiS**

- What do you think has worked well for your school?
- How has the programme been received by staff?
- Has the FFiS influence healthy eating in your school? What about wider outcomes?
- Have these interventions contributed to expected outcomes?

***Community beneficiaries: Pupils and parents/carers***

- What is pupils’ involvement (if any) in [initiatives discussed already]?
- What have pupils thought about these activities?
- Have [activities/intervention(s) mentioned] made a difference in pupils? [Probe: knowledge on healthy foods, attitudes towards healthy eating, eating practices?]
- Are they engaged with your school food culture? [Probe if influencing]
- Have you seen any other changes in pupils?
- What involvement (if any) do parents/carers have? If they are not involved, why do you think that is? How do you think they could be more involved?

**Part III: Recommendations and looking ahead**

- What’s the best way forward to improve any of these initiatives (if not already discussed)? [Probe: main issues raised throughout the interview].
- Are there lessons/shared learning from your experience that can be used to inform the wider FFiS?
- What could help to overcome these?
- What elements of the FFiS would you want to maintain (if any) at your school?
- What might make these more sustainable?
- Are there any future plans for other healthy food provision/nutrition programmes to be implemented at this school/ other?

**Part VI: Close**

- Do you have anything else you would like to add that we haven’t covered?

***Closing comments and provide a summary of the study***

| *We thank you for your interest and time in taking part if this study to help us better understand how children eat different types of food in their primary schools in Tower Hamlets. This is part of a programme where Tower Hamlets council is helping primary schools to improve their meals and support children to eat healthier. It is part of ActEarly, a wider initiative where universities, councils and communities come together to help improve children’s health.* |
| --- |

***Do you have any questions you would like to ask us about the discussion we just had or the study?***

***Remind them they can contact us at any time. Thank participant and discuss incentive for the school.***

Guide for Parents/carers (Topic guide 3)

| **Researcher - Please provide the following**:   - Introduce the FIGS Study. The interview is divided into two parts: 1) explore how food is consumed at the school: free school meals (FSMs), packed lunches, fruit and vegetable schemes, breakfast and afterschool clubs and 2) gather their views and experience of the Fantastic Food in Schools (FFiS) Programme. - Interviews will vary because of differing degrees of involvement staff will have with school food provision and the FFiS. - Emphasise that there is no right/wrong answer, this is a discussion to capture their views and experiences of school food provision and the FFiS. - **Informed consent**: Emphasise all information given will be treated in complete confidence – no individual will be identified in any reporting and their views will remain confidential, all transcriptions and extracts will use pseudonyms.   - Permission to record interview (audio) - **Duration:** ~1 hour - **Researcher:** The order of questions can change as the interview moves on. It is key to study the guide and be clear about questions in each section. - **Probing questions**: Probing questions follow from general, open questions, and then can support you to gather more detail and in case the interview loses ‘flow’. |
| --- |

**Part I: Introduction, FSMs and packed lunches**

- Start by asking them about their family: Who do they you live with? What about the child connected to the primary school? How are they finding the school?
- What type of school meal does your child usually have?
- Have they usually had this type of lunch or not? [If changed, explore when and why].
- Why did you decide on this type of lunch for your child? [Explore: dietary requirements, food preference, taste, financial, time pressures, cultural familiarity, control over what child eats, care].
- How does the school ask you to sign up for FSMs? [Per term/flexible?]
- How does your school tell you about their school food meal offers (FSMs/holiday school vouchers/breakfast-afterschool clubs, fruit and vegetable schemes)? Do you remember what messages they used?
- If [packed lunch], are you aware that the school offers FSMs to *all* children? If so, explore [if not covered] why they as parents do not take the offer.

**Parents’ meta-perspectives on school meals**

- Let’s now move on to talk about [child].
- What do they think of school dinners? Packed lunches? Others [mentioned above]?
- Does your child have friends/siblings who also have [packed lunch/school meal]? What do they like/dislike school foods? Do you know if other children think differently/not?

**Eating practices at home**

- Let’s talk now about what you eat at home: yesterday, what happened around dinner? Who cooked? Who sits and eats where? Is this your normal set-up?
- What about weekends? Does this change?
- What does [child] like/dislike to eat at home? What about at dinner? Other meals/ snacks?
- **UK cost-of-living crisis**: As you are probably aware, the UK is going through a cost-of-living crisis with millions of families struggling. May I ask if this has affected you/ your family?

**Part II: Fantastic Food in Schools Programme (FFiS)**

**Views and overall experience**

- Let’s now move on to talk about a programme your child’s [school] is doing to improve its food offer. Before FIGS, had you heard of the FFiS at your child’s school? [If not, please describe the FFiS]:

| *Over the next three years Tower Hamlets Council are running the FFiS to support schools to improve their FSM offer and implement a whole school approach to healthier eating. Your child’s primary school is part of this programme.* |
| --- |

- Do you know of any [interventions/activities] the school is running as part of this? If yes, what do you think of these? If not, why do you think you haven’t?
- Has your child mentioned any activities?
- What is your child’s involvement (if any) in [initiatives discussed]?

**Community beneficiaries: Pupils and parents/carers**

- If [activities mentioned above], have these made a difference to your child? [Probe: knowledge on healthy foods, attitudes towards healthy eating, eating practices?]
- Are they engaged with their school food culture? [Probe if influencing]
- Have you seen any other changes in your child?
- What involvement (if any) have you had in [activities discussed above]?
- If not involved, why do you think that is? Would they like to be more involved?
- What do you think about what the school is doing in FFiS? Should it to continue?
- What’s the best way forward to improve any of these (if not already discussed)? [Probe: main issues raised throughout the interview].

**Part III: Close**

- Do you have anything else you would like to add that we haven’t covered?

***Closing comments and provide a summary of the study***

| *We thank you for your interest and time in taking part if this study to help us better understand how children eat different types of food in their primary schools in Tower Hamlets. This is part of a programme where Tower Hamlets council is helping primary schools to improve their meals and support children to eat healthier It is part of ActEarly, a wider initiative where universities, councils and communities come together to help improve children’s health.* |
| --- |

***Do you have any questions you would like to ask us about the discussion we just had or the study?***

***Remind them they can contact us at any time. Thank participant and provide incentive.***

Guide with Children (Topic guide 4)

| **Welcome AND INTROS!**  Thank you for coming to the FIGS ‘table’. Researcher: Introduce yourself and FIGS. Today we are going to talk about food, what you like/not so much and what you eat here [Primary school] and at home. This is not a test! This is about you! We want to hear from you, the children experts from [Primary school]. Do we know what “expert” means? [Explain].  ***Guidance notes (Stoilova, et al., 2016):***   - *Enable an environment that encourages informal discussion and spontaneous participation, creating a trusting atmosphere.* - *Encourage the children to feel in control and understand the rules: this group space will be about: respect, rights, safety and wellbeing. Confidentiality and ethics of recording. Accommodate the discussion to the children’s ages and years and pay attention to diversity and inequalities (i.e., consider gender, ethnicity, language, confidence levels, additional needs). Continue monitoring throughout.* - *Giving opportunities for questions throughout.* - **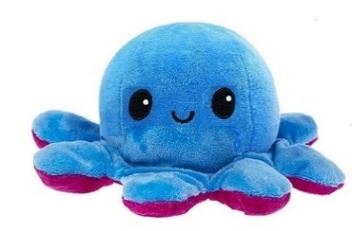***If any child feels uncomfortable stop and check, remind them they can: stop, not answer, not do the task or leave anytime (continue monitoring, offer again, if necessary).*   Pulpy is going to help us – Pulpy is a friendly octopus (stuffed toy)  that we will hold so each one talks when it's their turn.  Pulpy belongs to a girl who goes to a primary school like you,  she is helping us today by sharing Pulpy.   - Ask children to introduce themselves: when you have Pulpy - Please tell us what name you want to use with us today (you can choose any name!), your year and your favourite TV show/film/book. I’ll go first [introduce yourself]. |
| --- |

**Part I: School meals and packed lunches**

-
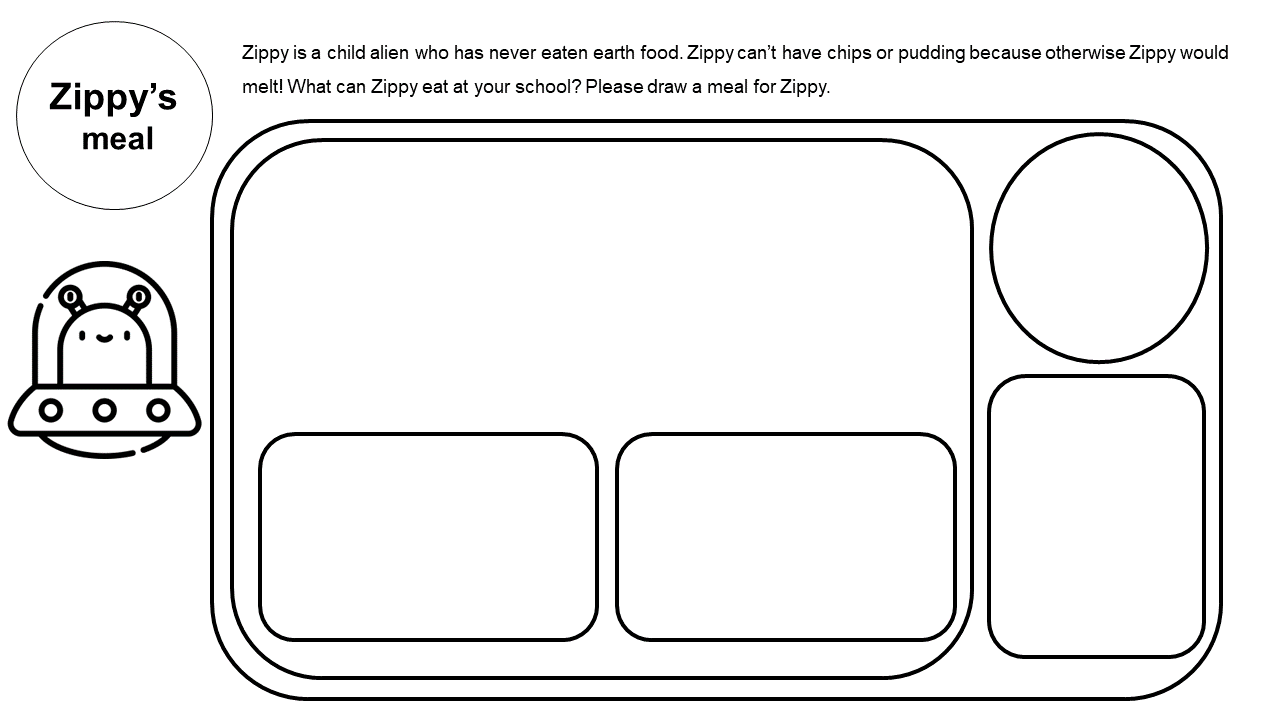
To start: ‘what’s the first thing that pops into your head when you think about the word yummy?’

| • Activity 1: Short story on healthier options and food preferences at school – please distribute the drawings to the children.  *Zippy is a child alien who has never eaten earth food but cannot eat pudding or chips (because they would otherwise melt!). Let’s pretend Zippy came here to [Primary school] and you had to show them around for the day. It is lunch time, can you help Zippy and draw him something they could eat at lunch?* |
| --- |

- Give the children some minutes. Let’s have a look at your drawings – can you tell me what you have drawn for Zippy?
  - [Explore: food preference, taste, cultural familiarity, dislikes]
- How do these compare to [packed lunch/school dinner]?
- What type of lunches do you have? Packed lunch or school dinners? [Explore: why they have this type of lunch/ Have this always been the case? If changed, explore when and why]. What about your class/friends?
- What’s a popular dinner at school? Is there a popular day?
- What about at other times/breaks? [Explore breakfast, afterschool clubs, snack times]
- Will Zippy find things changed at school with food [from last year/term]?
- What would be good/not so much?
- If Zippy asks about [FFiS: new things the school is doing around lunch and other meals]? What do we tell Zippy?
- Have you done anything? Cooking classes? Healthy foods? [Explore whole approaches to food, school food culture]

**
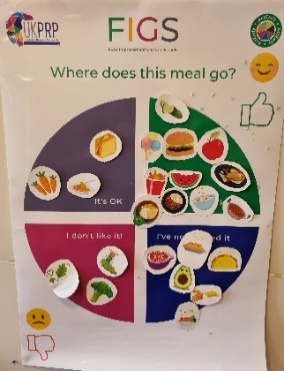
**

| *Activity 3: ‘Pin the meal’ on the graph’ ask the children to rate school meals in a cartesian plane with quadrants: ‘not for me’, ‘it’s alright’, ‘I want to have it again’.* |
| --- |

- Explore the choices the children make as they carry out this activity.

| *Activity 3a: ‘Food challenge’: explore what would get children (non-users) to have school meals by asking them to choose between some of the food stickers (curry vs pasta; broccoli vs carrot) placed on the quadrants.* |
| --- |


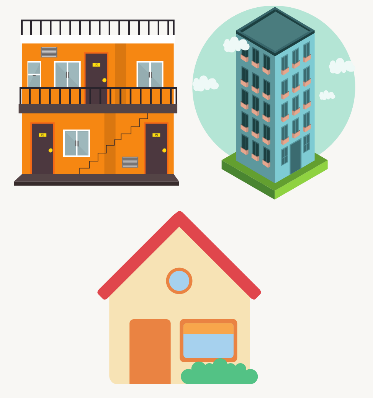
**Part II: Eating practices at home**

- Let’s close our eyes and [pretend] we travel back in time and we’re back at home yesterday and it’s around the time when you eat before bedtime.
- What did you eat? Did you like it/not?
- What happened at dinner? Who cooked/gave you food?
- What about weekends? Does this change?
- What do you like/dislike about the food [cook] makes for you at home/with your family?

**Part III: Close**

- Is there anything you would like to say to your school/teachers? Parents/carers about food/things we’ve talked about today?
- Do you have anything else you would like to say?

***Closing comments and provide a summary of the study***

***Do you have any questions about this group chat?***

***Remind them their parents/carers/teachers can contact us at any time.***

***Thank the children and provide incentive.***

***
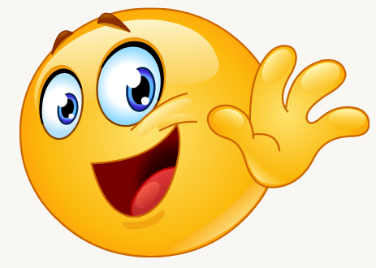
***

Observation template

**Researcher: _____________ Date: ____________ School: ________________**

**Time spent in school: _______ hrs ______ minutes**

| **Setting** | Describe the school: surrounding area, internal environment (size of school, pupils, staff, facilities, etc) |
| --- | --- |
|  |  |
| **School meal hall environment** | Describe the layout and food (queuing, food on offer, sensorial input, seating arrangements, time constraints, supporting staff) |
|  | Describe interactions (between teachers, kitchen and other staff and pupils, peers) |
| **Intervention/activities as part of the programme** | Please describe each intervention(s) or activity: [TastEd, Parent taster, Cook-a-thon, other] been observed: what is been delivered and where, who are involved, who is the activity for (children/parents/staff), how many are present, what is working well, what can be improved, how is it received)  Intervention/Activity 1:  Intervention/Activity 2: |
| **Daily reflexions** | What else happened today? What did you observe? Did anything catch your attention/surprise you? |
|  | Who did you talk to? |
|  | What worked well today? What could have gone better? |
| **Context of data collection** | Provide a description of the data collection methods (if applicable):   1. Interviews: 2. Mini-groups: |
|  | Instruments (did they work well? Did the questions work in practice? How did you find the structure and flow? Do you have any suggestions?) |
|  | Interview/group dynamics (trust, rapport, tensions) |
|  | What worked well?  Were there any issues? |
| **Reflexivity and positioning** | How do you situate yourself in the interview/group encounter? In the wider school environment?  How did you feel today? |
| **Other** | Any other observations/reflexions? |
